# Mask-Wearing and Handwashing Behaviors of Chinese Rural Residents during the Pandemic of COVID-19: A Cross-Sectional Survey

**DOI:** 10.3390/ijerph20010779

**Published:** 2022-12-31

**Authors:** Zhiyuan Zheng, Chengyao Liang, Zhuoyang Li, Yugao Wu, Baixue Lin, Jing Fang

**Affiliations:** Institute for Health Sciences, Kunming Medical University, Kunming 650500, China

**Keywords:** COVID-19, mask-wearing, handwashing, influencing factor, rural residents

## Abstract

Objective: To understand mask-wearing and handwashing behaviors of Chinese rural residents during the COVID-19 pandemic and to analyze the associated factors. Methods: This study used a multi-stage random sampling method to conduct a cross-sectional questionnaire survey during the period of July to December of 2021, in six counties located in Shandong, Shanxi, and Yunnan provinces representing the eastern, central, and western regions of China, respectively. A total of 3864 villagers were surveyed with a questionnaire, and 3832 valid questionnaires were finally analyzed. Descriptive statistics and logistic regression analysis were used for statistical analysis. Results: Around ninety-four percent (93.6%) of rural residents reported mask-wearing during the COVID-19 pandemic, but only 44.5% of them could replace masks in time. Multivariate logistic regression analysis showed that those who were female, aged 15–59, had an education level of high school and above, were divorced/widowed, worked as farmers (workers), or were rural residents in Shandong Province were more likely to wear masks. Furthermore, those who were female, aged 15–59, had an education level of high school and above, were unmarried and married, were business and service workers, or were rural residents in Shandong and Shanxi Province replaced masks more timely. Around seventy percent (69.7%) of rural residents reported using soap when washing their hands, but only 38.0% of rural residents could wash their hands properly. Multivariate logistic regression analysis showed that rural residents who were aged 35–59, had an education of high school and above, or lived in Shandong Province and Shanxi Province were more likely to wash their hands with soap. Those who were aged 15–59, had an education of high school and above, worked as farmers (workers), were employees of governmental departments and retirees, were business and service workers, or were students had higher proper handwashing rates. Conclusions: During the COVID-19 pandemic, the proportion of Chinese rural residents wearing masks reached 93.6%, but only 44.5% were able to replace masks in time, gender, age, education level, marital status, occupation, and living place had an impact on mask-wearing. The proportion of Chinese rural residents who could wash hands with soap reached 69.7%, but only 38.0% could wash their hands properly. Age and education level were influencing factors for both washing-hand with soap and proper handwashing.

## 1. Background

The coronavirus 2019 (COVID-19) pandemic is still ongoing. The main transmission routes of COVID-19 are respiratory droplet and contact transmissions [1] which led to its global rapid spread due to its highly infectious nature. As of 18 September 2022, over 609 million confirmed cases and 6.5 million deaths have been reported globally [2]. The virus of severe acute respiratory syndrome coronavirus 2 (SARS-CoV-2) constantly mutates, creating what has been termed the new normal characterized by dynamic changes and repeated invasion of SARS-CoV-2 [3]. Under this new normal, China has gradually formed a “Timely Clearing Epidemics” prevention and control strategy aiming at “prevention infection transmitted from abroad and infection rebounded from domestic” [4,5]. This strategy successfully kept the COVID-19 pandemic at a low level in China before the end of 2022. However, studies have shown that there is no effective treatment for COVID-19 [6], and achieving zero infections remains challenging. According to the report of the National Health Commission of China, since 29 September 2022 provinces across the country have reported new local infections; indeed community transmission and epidemic spillover have not been completely stopped, and the prevention and control situation is severe and complex [7]. Therefore, prevention remains a very effective measure to avoid COVID-19 infection.

Respiratory droplet is one of the main transmission routes of SARS-CoV-2. Daily face-to-face conversation, breathing, and coughing may produce droplets, which may enter susceptible mucosal surfaces within a certain distance and SARS-CoV-2 can be transmitted from the respiratory tract through droplets [8]. Exhaled droplets can further form aerosols in the air with diameters between 0.001 and 100 μm [9]. With the help of airflow, aerosols can spread to a wider range, and SARS-CoV-2 can survive in aerosols for several hours and be infectious [10]. Wearing masks not only minimizes the number of viruses emitted into the atmosphere by virus carriers but also slows down the spread of droplets and aerosols generated by conversation, breathing, and coughing, to reduce the spread area. Secondly, wearing masks can protect healthy people from inhaling viruses contained in droplets and aerosols in the air so as to cut off virus transmission [11,12,13]. Therefore, during the COVID-19 pandemic, masks became an essential personal protective equipment [14]. The World Health Organization also issued guidelines on the necessity of wearing masks [15], and studies have shown that mask-wearing is an effective measure to prevent COVID-19 infection [16,17,18].

As a basic personal protection measure, handwashing is regarded as one of the most effective means to prevent infectious diseases, and has always played an important role in the prevention of infectious diseases [19,20]. Fung et al. compiled studies on the effectiveness of handwashing behaviors in transmission intervention of SARS coronavirus during the SARS epidemic, through a comprehensive analysis of data from 10 epidemiological studies. The analysis found that handwashing plays an important role in the prevention and control of SARS virus transmission [21]. In addition, Cairncross’s study showed that handwashing is an easy and important measure to reduce diarrhea and reduce acute respiratory infections in children [22]. Stephen et al. found that the promotion of handwashing was one of the most widespread and effective measures in the prevention and control of cholera epidemics in Asia [23]. Research showed that SARS-CoV-2 can survive on human skin for more than 9 h [24], so skin-to-skin transmission is also considered as another major route of SARS-CoV-2 transmission [25]. Therefore, handwashing has also become an important measure to prevent COVID-19, studies have thus proved that handwashing can indeed reduce the risk of COVID-19 infection [26,27]. The World Health Organization’s guidelines also states the important role of handwashing in the prevention of COVID-19 transmission [28].

COVID-19 has prevailed worldwide for three years and might continue to for an unknown length of time. Mask-wearing and handwashing have been widely recommended as the personal preventive measure to prevent and control the pandemic. The Chinese government issued policy documents and guidelines asking people to wear masks and wash hands during the COVID-19 pandemic [29,30], but the compliance of the public is unknown, especially the compliance of rural residents who account for 36.11% of China’s population [31]. Therefore, this study aims to investigate the mask-wearing and handwashing behaviors of Chinese rural residents as well as influencing factors during the COVID-19 pandemic in order to inform the plan and design more-targeted interventions for the prevention and control of COVID-19.

## 2. Methods

### 2.1. Study Design

This study is a cross-sectional survey using a multi-stage random sampling method. Firstly, Shandong Province, Shanxi Province, and Yunnan Province were randomly selected as sample provinces to represent provinces located in the eastern, central, and western regions of China, and two counties were randomly selected as sample counties in each of the three sampled provinces. Namely, Changle County in Weifang City and Shan County in Heze City, Shandong Province; Zhongyang County and Lan County in Lvliang City, Shanxi Province; and Yao’an County in Chuxiong Yi Autonomous Prefecture and Zhenyuan Country in Pu’er City, Yunnan Province. Secondly, two townships in each sampled county were randomly selected as sample townships. Thirdly, three villages in each of the 12 sampled townships were randomly selected as sampling villages. Finally, a simple random sampling method was used to randomly select residents over 15 years of age who have resided in the village for more than 6 months.

### 2.2. Sampling and Sample Size

The health literacy level of Chinese rural residents in 2020 is 20.02% [32], we then used P = 0.2002, supposing δ = P × 20% = 0.04004, μ(1 − α/2) = 1.96, design efficiency = 1.5, according to the formula: $n=\frac{\mu_{\left(1-\alpha /2\right)}^{2}\times P\left(1-P\right)}{\delta^{2}}\times \mathrm{deff}$, *n* ≈ 576 is obtained for each county. Considering the possibility of invalid responses, the actual sample size was expanded by 5%, therefore 605 people should be investigated in each sampled county.

### 2.3. Data Collection

The self-designed questionnaire was used by field investigators to conduct a face-to-face interview with the respondents. Prior to the survey, all field investigators who are graduate students and teachers from the School of Public Health of Kunming Medical University, were strictly trained to ensure that they perform the survey. According to unified and standardized procedures. During the field survey, survey mentors checked every completed questionnaire to identify any missing item or mistake and returned the questionnaire to the investigator for correction whenever a problem was identified. Collected data included gender, age, education level, marital status, occupation, living place (province), mask-wearing, replacing behaviors covering whether to wear a mask when going out and how long for using each mask, handwashing behavior covering under what circumstances they washed hands, whether they used running water to wash hands, whether they washed hands with soap, and whether they washed hands for 20S and above. Except for the question of how long each mask is used for, all other questions had three answer options: always, sometimes, and never.

According to the “Guidelines for the Public to Proper Wear Face Mask” by the National Health Commission of the People’s Republic of China [29], a single mask should be replaced after being worn for four hours. Therefore, we define the timely replacement of a mask as it being worn for less than four hours, thus defining untimely mask replacement as being a single mask worn for more than four hours.

According to the WHO guidelines on hand hygiene [33,34], proper hand washing should be performed before eating, after going to the toilet, after working, and after going to the hospital and contacting patients. In addition, it should entail the use of running water when washing hands, using soap when washing hands, and washing hands for at least 20 s. Accordingly, those questions about handwashing in the survey were combined into one indicator: whether to wash hands properly. Respondents were categorized as washing hands properly only if they answered “always” to all the above-mentioned questions about handwashing.

In total, 3864 questionnaires were obtained and 32 unqualified questionnaires were excluded from analysis. The final study sample size was 3832.

### 2.4. Statistical Analysis

All data were analyzed by IBM SPSS Statistics version 25.0. Classification variables are described as numbers and proportions (%). The Chi-squared test was used to compare the regional/province differences in demographic variables, and univariate logistic regression was used to analyze the mask-wearing and handwashing behaviors of rural residents with different demographic characteristics. Variables with *p*-values < 0.05 in the univariate analysis were included in the final multivariate analysis to adjust for potential confounders. The results of the regression analysis are presented with odds ratios (ORs) and 95% confidence intervals (CIs) as well as coefficiencies β. A *p*-value < 0.05 was considered to be statistically significant.

## 3. Results

### 3.1. Characteristics of Participants

A total of 3832 valid questionnaires were obtained in this study, of which female accounted for 50.5% slightly greater than the male; respondents aged 15–34, 35–59, and ≥60 years old accounted for 18.0%, 47.3%, and 34.7% of the total sample; the largest number of respondents had middle school education, accounting for 35.0%; most respondents’ marital status was married accounting for 86.5%; more than half of the respondents’ occupations were farmers (workers) accounting for 56.45%; the distribution of respondents in the three sampled provinces was almost even with 33.0% in Shandong Province, 33.1% in Shanxi Province, and 33.9% in Yunnan Province. The different distributions of demographic variables including age (χ^2^ = 59.825, *p* < 0.001), education level (χ^2^ = 106.336, *p* < 0.001), marital status (χ^2^ = 10.970, *p* = 0.027), and occupation (χ^2^ = 757.796, *p* < 0.001) were statistically significant among the three provinces, with Shandong province having the greatest group of people aged ≥60 who are illiterate or have little literacy. Whereas, Shanxi have the greatest group of high school and above, Yunnan have the smallest group of unmarried people, and Shanxi have the biggest group of people who are unemployed. Details about the demographic characteristics of the survey respondents are shown in Table 1.

### 3.2. Prevalence Rate of Mask-Wearing and Its Influencing Factors

Among all 3832 respondents, 3586 reported always wearing a mask, 101 reported sometimes wearing a mask, and 145 reported never wearing a mask during the COVID-19 pandemic. Those who reported always wearing a mask accounted for 93.6% (Table 2).

The multivariate logistic regression analysis results showed that females compared with male (OR = 0.379, *p* < 0.001); people aged 15–34 (OR = 2.871, *p* = 0.001) and aged 35–59 (OR = 1.468, *p* = 0.018) compared with aged ≥ 60; people with aneducational level of high school and above compared with illiterate or those withlittle literacy (OR = 0.525, *p* = 0.013); people with a marital status of divorced/widowed compared with unmarried (OR = 0.345, *p* = 0.003); people with an a farming (workers) occupation (OR = 1.695, *p* = 0.002) compared with those who are unemployed; or people who are rural residents of Shandong province (OR = 1.569, *p* = 0.022) compared with Yunnan province, were more likely to wear masks (Table 3).

### 3.3. Prevalence Rate of Timely Mask Replacement and Its Influencing Factors

Among all 3586 survey respondents who wore masks, in total 1594 were able to replace their masks in time, accounting for 44.5%.

The multivariate logistic regression analysis results showed that females compared with males (OR = 0.814, *p* = 0.009); people aged 15–34 (OR = 2.199, *p* < 0.001) and aged 35–59 (OR = 1.660, *p* < 0.001) compared with those aged ≥ 60; people with an educational level of high school and above compared with middle school (OR = 0.765, *p* = 0.023), primary school (OR = 0.657, *p* = 0.002), and illiterate levels or have little literacy (OR = 0.543, *p* < 0.001); people with their marital status as unmarried (OR = 2.023, *p* = 0.007) and married (OR = 1.555, *p* = 0.004) compared with divorced/widowed; people with a business and service workers occupation (OR = 1.489, *p* = 0.003) compared with those who are unemployed; or people who are rural residents in Shandong province (OR = 3.948, *p* < 0.001) and Shanxi province (OR = 1.314, *p* = 0.003) compared with Yunnan province, were more likely to replace their masks in time (Table 4).

### 3.4. Prevalence Rate of Handwashing with Soap and Its Influencing Factors

Among all 3832 respondents, 2672 reported always washing-hands with soap, 911 reported sometimes washing-hands with soap, and 249 reported never handwashing with soap. Those who reported always washing their hands with soap accounted for 69.7% (Table 5).

The multivariate logistic regression analysis results showed that people aged 35–59 (OR = 1.408, *p* < 0.001) compared with those aged ≥ 60; people with an educational level of high school and above compared with primary school (OR = 0.727, *p* = 0.022) and illiterate levels or have little literacy (OR = 0.630, *p* = 0.001); or people who are rural residents in Shandong province (OR = 1.799, *p* < 0.001) and Shanxi province (OR = 2.331, *p* < 0.001) compared with Yunnan province, were more likely to wash their hands with soap (Table 6).

### 3.5. Prevalence Rate of Proper Handwashing and Its Influencing Factors

According to the WHO’s guidelines on hand hygiene, among all 3832 respondents, 1457 could wash their hands properly, which was counted with survey respondents who answered “always” to all the seven handwashing questions, accounting for 38.0%. The details about the seven handwashing questions and the answers given by the survey respondents are shown in Table 5.

The multivariate logistic results showed that people aged 15–34 (OR = 1.284, *p* = 0.035) and aged 35–59 (OR = 1.356, *p* < 0.001) compared with those aged ≥ 60; people with an educational level of high school and above compared with illiterate levels or people have little literacy (OR = 0.554, *p* < 0.001) and primary school (OR = 0.635, *p* < 0.001); people who have a farming (workers) occupation as (OR = 1.245, *p* = 0.011), employees of governmental departments and retirees (OR = 2.210, *p* < 0.001), business and service workers (OR = 1.719, *p* < 0.001), or students (OR = 2.362, *p* = 0.004) compared with those who are unemployed, were more likely to wash their hands properly (Table 7).

## 4. Discussion

Respiratory droplet and contact are the main routes of COVID-19 transmission [35]. Under the current “new normal” of the pandemic, people need to properly wear masks and wash their hands to prevent the disease [36]. In our study, we found that the prevalence rate of mask-wearing among Chinese rural residents during the pandemic of COVID-19 was 93.6%, which is higher and similar to the results of other studies in China [37,38], and it was also higher than the result of 84.6% reported by Rader et al. through a cross-sectional survey in the United States in the second half of 2020 [39]. The results of multivariate logistic regression analysis showed that rural residents who were female, aged 15–59, or had an education level of high school and above, were more likely to wear masks, which is consistent with the results of other studies [40,41]. This may be related to the fact that these groups have better hygienic habits and awareness of self-protection in their daily lives, and they have better acceptance and compliance with the government’s policy and guidelines on disease prevention. Notably, COVID-19 causes higher mortality in the elderly population [42]; however, our findings showed that the group of people aged 60 and above had a lower proportion of mask-wearing. Our findings also showed that divorced/widowed individuals compared with the unmarried were more likely to wear masks and the underlying reasons were unknown, probably due to sample bias, which needs further study in the future. Farmers (workers) were more likely to wear masks than the unemployed, which may be related to the fact that they are required to wear masks at workplaces. Rural residents in Shandong Province were more likely to wear masks, which may be related to the fact that the health literacy in the eastern region is better than that in the central and western regions [43].

Wearing a mask can effectively reduce virus infection, but wearing a single mask for a long time will not only make its protective performance greatly reduced and increase the risk of respiratory infections, but also may cause bacteria to grow in the inner layer of the mask. This is due to breathing in water vapor which makes breathing resistance increase and thus affects breathing, hence the mask should be replaced in time to achieve the best protection effect [44,45]. Our study found that only 44.5% of the rural residents could replace their masks in time. Those who were male, aged ≥ 60, or had an education of middle school and below, were less likely to replace their masks in time, which is consistent with the results of the study in Shanghai, China [46]. This may be caused by their limited knowledge about COVID-19 prevention and limited accessibility to masks; however, the underlying reasons need further study in the future. The divorced/widowed group had a higher proportion of mask-wearing than the unmarried; however, they were less likely to replace masks than the unmarried and married, the reasons for this need further study. Business and service workers were timelier in replacing their masks than the unemployed, which may be related to requirements and more-intensified COVID-19 prevention knowledge delivered at workplaces. The timelier replacement of masks by rural residents in Shandong Province and Shanxi Province may be due to the more effective publicity and education taken by the local governments and also, the better accessibility of masks in economically more developed regions. To summarize, the female, the younger, the unmarried, the more educated, and the rural residents of Shandong Province had better mask-wearing and timely-replacing behavior, which may be related to their better hygiene habits, and greater awareness of self-protection.

Handwashing is also a very important measure for the prevention of COVID-19, and studies have shown that handwashing reduces the risk of COVID-19 infection [26,47]. Earlier studies have also found that handwashing reduces the transmission of respiratory viruses by 45–55% [48]. In particular, handwashing with soap is one of the cheapest and most effective ways of preventing diseases [35,49]. In our study, we found that 69.7% of the rural residents could wash their hands with soap, which was significantly higher than the 25.1% reported by Tao et al. in a survey conducted in five Chinese provinces in 2013 prior to the COVID-19 pandemic [50]. This implies that the COVID-19 pandemic increases the prevalence rate of handwashing with soap among Chinese rural residents. Our multivariate logistic regression analysis result showed that those who were aged 35–59 years, had an with education level of high school and above, were more likely to use soap when washing their hands, which is consistent with the results reported by studies from other countries [51,52]. This may be related to the better hygiene practices of those groups. Rural residents in Shandong and Shanxi provinces were more likely to use soap when washing their hands than their counterparts in Yunnan, probably due to the better economic condition and health knowledge of rural residents in Shandong and Shanxi provinces.

Although handwashing is a relatively inexpensive and effective measure that can be widely used for personal protection, it is very difficult to maintain a high rate of proper handwashing. In our study, we found that only 38.0% of the rural residents could wash their hands properly. By further analyzing their handwashing behavior and influencing factors, we found that people aged 15–59, those with an education level of high school and above, those with a farming (workers) occupation, those who are employees of governmental departments and retirees, those who are business and service workers, and those who are students have a higher probability of proper handwashing, which may be related to the higher health literacy of those groups, which is consistent with the studies from China and Iran [41,43]. Health education and outreach interventions of COVID-19 prevention should pay more attention to targeting rural residents who are male, elderly, have a lower education level, and those who are unemployed for proper mask-wearing and handwashing behaviors to enable them to understand the importance of these behaviors for disease prevention, so as to forge a better prevention effect in the “new normal” era of COVID-19 pandemic. Better handwashing with soap and proper handwashing behavior among younger, more educated, and rural residents may be related to their higher receptivity to external information and ability to execute guidelines.

## 5. Limitation

This study has some limitations. Firstly, it is a cross-sectional survey, and because of the inherent nature of this research method, a causal relationship cannot be established. Secondly, this study selected Shandong, Shanxi, and Yunnan as sample provinces for the eastern, central, and western regions of China, and a selection of three provinces may be too limited to represent the whole of China given its vast area, diverse ethnic groups, and socioeconomic development levels. Future study is needed to cover more provinces in China so as to obtain a more comprehensive understanding of mask-wearing and handwashing behaviors of rural residents in China.

## 6. Conclusions

Proper mask-wearing and handwashing behaviors are important protective methods to prevent the spread of COVID-19. We used a questionnaire to investigate mask-wearing and handwashing behaviors of Chinese rural residents during the COVID-19 pandemic. Our survey showed that although the proportion of Chinese rural residents wearing masks during the COVID-19 pandemic reached 93.6%, only 44.5% of them can replace masks in time. Gender, age, education level, marital status, occupation, and living place (province) are the common influencing factors for both mask-wearing and replacement. The proportion of Chinese rural residents who used soap to wash hands during the pandemic of COVID-19 increased significantly compared with the pre-pandemic period, reaching 69.7%, but only 38.0% were able to wash hands properly, with age and education level being the common influencing factors for both washing hand with soap and proper handwashing. Health education and outreach interventions of COVID-19 prevention in rural China should pay more attention to target rural residents who are male, elderly, with a lower education level, and those who are unemployed for proper mask-wearing and handwashing behaviors, so as to have a better prevention effect in the “new normal” era of the COVID-19 pandemic.

## Figures and Tables

**Table 1:** Characteristics of 3832 rural residents.

Variables	Shandong n (%)	Shanxi n (%)	Yunnan n (%)	Total n (%)	χ^2^	*p*
**Gender**						
Male	600 (47.4)	633 (50.0)	665 (51.2)	1898 (49.5)	3.843	0.146
Female	666 (52.6)	634 (50.0)	634 (48.8)	1934 (50.5)		
**Age (years)**						
15–34	153 (12.1)	262 (20.7)	274 (21.1)	689 (18.0)	59.825	<0.001
35–59	601 (47.5)	573 (45.2)	638 (49.1)	1812 (47.3)		
≥60	512 (40.4)	432 (34.1)	387 (29.8)	1331 (34.7)		
**Education level**						
Illiterate or have little literacy	442 (34.9)	237 (18.7)	340 (26.2)	1019 (26.6)	106.336	<0.001
Primary school	221 (17.5)	349 (27.5)	320 (24.6)	890 (23.2)		
Middle school	427 (33.7)	447 (35.3)	466 (35.9)	1340 (35.0)		
High school and above	176 (13.9)	234 (18.5)	173 (13.3)	583 (15.2)		
**Marital status**						
Unmarried	82 (6.5)	72 (5.7)	49 (3.8)	203 (5.3)	10.970	0.027
Married	1090 (86.1)	1087 (85.8)	1139 (87.7)	3316 (86.5)		
Divorced/widowed	94 (7.4)	108 (8.5)	111 (8.5)	313 (8.2)		
**Occupation**						
Farmer (worker)	979 (77.3)	454 (35.8)	730 (56.2)	2163 (56.4)	757.796	<0.001
Employees of governmental departments and retirees	56 (4.4)	78 (6.2)	59 (4.5)	193 (5.0)		
Business and service workers	142 (11.2)	137 (10.8)	187 (14.4)	466 (12.2)		
Students	57 (4.5)	14 (1.1)	7 (0.5)	78 (2.0)		
Unemployed	32 (2.5)	584 (46.1)	316 (24.3)	932 (24.3)		

**Table 2:** Mask-wearing by 3832 rural residents.

Frequency	Shandong Province n (%)	Shanxi Province n (%)	Yunnan Province n (%)	Total n (%)
Always	1214 (95.9)	1159 (91.5)	1213 (93.4)	3586 (93.6)
Sometimes	25 (2.0)	40 (3.2)	36 (2.8)	101 (2.6)
Never	27 (2.1)	68 (5.4)	50 (3.8)	145 (3.8)

**Table 3:** Logistic regression analysis for influencing factors of mask-wearing (n = 3832).

Variables	Total n (%)	Mask-Wearing n (%)	Univariate		Multivariate		
			OR (95% CI)	*p*-Value	*β*	OR (95% CI)	*p*-Value
**Gender**							
Male	1898 (49.5)	1729 (91.0)	0.424 (0.321–0.560)	<0.001	–0.969	0.379 (0.279–0.516)	<0.001
Female	1934 (50.5)	1857 (96.0)	1			1	
**Age (years)**							
15–34	689 (18.0)	664 (96.3)	2.924 (1.887–4.531)	<0.001	1.055	2.871 (1.572–5.245)	0.001
35–59	1812 (47.3)	1723 (95.0)	2.131 (1.613–2.816)	<0.001	0.384	1.468 (1.069–2.014)	0.018
≥60	1331 (34.7)	1199 (90.0)	1			1	
**Education level**							
Illiterate or have little literacy	1019 (26.6)	921 (90.3)	0.564 (0.375–0.848)	0.006	–0.645	0.525 (0.316–0.872)	0.013
Primary school	890 (23.2)	832 (93.4)	0.861 (0.554–1.337)	0.505	0.004	1.004 (0.600–1.679)	0.989
Middle school	1340 (35)	1283 (95.7)	1.351 (0.870–2.097)	0.181	0.206	1.229 (0.752–2.007)	0.410
High school and above	583 (15.2)	550 (94.3)	1			1	
**Marital status**							
Unmarried	203 (5.3)	179 (88.1)	0.969 (0.559–1.679)	0.912	–1.064	0.345 (0.172–0.693)	0.003
Married	3316 (86.5)	3130 (94.3)	2.187 (1.499–3.190)	<0.001	0.312	1.367 (0.912–2.048)	0.130
Divorced/widowed	313 (8.2)	277 (88.4)	1			1	
**Occupation**							
Farmer (worker)	2163 (56.4)	2048 (94.6)	1.950 (1.466–2.595)	<0.001	0.528	1.695 (1.205–2.386)	0.002
Employees of governmental departments and retirees	193 (5.0)	181 (93.7)	1.652 (0.886–3.079)	0.114	0.410	1.507 (0.763–2.977)	0.237
Business and service workers	466 (12.2)	443 (95.0)	2.110 (1.317–3.379)	0.002	0.319	1.375 (0.815–2.320)	0.232
Students	78 (2.0)	74 (94.8)	2.026 (0.724–5.669)	0.179	0.759	2.136 (0.634–7.194)	0.221
Unemployed	932 (24.3)	840 (90.1)	1			1	
**Province**							
Shandong	1266 (33.0)	1215 (95.9)	1.689 (1.184–2.410)	0.004	0.450	1.569 (1.068–2.304)	0.022
Shanxi	1267 (33.1)	1158 (91.3)	0.753 (0.561–1.011)	0.059	–0.233	0.792 (0.581–1.080)	0.141
Yunnan	1299 (33.9)	1213 (93.3)	1			1	

**Table 4:** Logistic regression analysis for influencing factors on mask replacement (n = 3586).

Variables	Total n (%)	Replacing Masks in Time n (%)	Univariate		Multivariate		
			OR (95% CI)	*p*-Value	*β*	OR (95% CI)	*p*-Value
**Gender**							
Male	1729 (48.2)	725 (41.9)	0.823 (0.721–0.939)	0.004	–0.206	0.814 (0.698–0.949)	0.009
Female	1857 (51.8)	869 (46.8)	1			1	
**Age (years)**							
15–34	664 (18.5)	373 (56.2)	2.529 (2.082–3.072)	<0.001	0.788	2.199 (1.705–2.836)	<0.001
35–59	1723 (48.0)	819 (47.5)	1.794 (1.540–2.090)	<0.001	0.507	1.660 (1.387–1.988)	<0.001
≥60	1199 (33.4)	402 (33.5)	1			1	
**Education level**							
Illiterate or have little literacy	921 (25.7)	340 (36.9)	0.588 (0.475–0.729)	<0.001	–0.611	0.543 (0.413–0.714)	<0.001
Primary school	832 (23.2)	324 (38.9)	0.640 (0.515–0.796)	<0.001	–0.419	0.657 (0.507–0.852)	0.002
Middle school	1283 (35.8)	617 (48.1)	0.930 (0.761–1.135)	0.475	–0.268	0.765 (0.607–0.963)	0.023
High school and above	550 (15.3)	313 (56.9)	1			1	
**Marital status**							
Unmarried	179 (5.0)	117 (65.4)	5.177 (3.446–7.777)	<0.001	0.705	2.023 (1.210–3.382)	0.007
Married	3130 (87.3)	1403 (44.8)	2.229 (1.692–2.935)	<0.001	0.442	1.555 (1.153–2.099)	0.004
Divorced/widowed	277 (7.7)	74 (26.7)	1			1	
**Occupation**							
Farmer (worker)	2048 (57.1)	931 (45.5)	1.703 (1.440–2.015)	<0.001	0.131	1.140 (0.928–1.400)	0.212
Employees of governmental departments and retirees	181 (5.0)	86 (47.5)	2.552 (2.015–3.232)	<0.001	0.088	1.092 (0.752–1.585)	0.645
Business and service workers	443 (12.4)	246 (55.5)	1.850 (1.336–2.561)	<0.001	0.398	1.489 (1.144–1.938)	0.003
Students	74 (2.1)	55 (74.3)	5.915 (3.443–10.162)	<0.001	0.087	1.091 (0.547–2.177)	0.804
Unemployed	840 (23.4)	276 (32.9)	1			1	
**Province**							
Shandong	1215 (33.9)	756 (62.2)	3.380 (2.861–3.994)	<0.001	1.373	3.948 (3.284–4.474)	<0.001
Shanxi	1158 (32.3)	440 (38.0)	1.253 (1.059–1.483)	0.009	0.273	1.314 (1.097–1.574)	0.003
Yunnan	1213 (33.8)	398 (32.8)	1			1	

**Table 5:** Handwashing by 3832 rural residents.

Projects	Shandong			Shanxi			Yunnan			Total		
	Always n (%)	Sometimes n (%)	Never n (%)	Always n (%)	Sometimes n (%)	Never n (%)	Always n (%)	Sometimes n (%)	Never n (%)	Always n (%)	Sometimes n (%)	Never n (%)
Washing hands with soap	913 (72.1)	272 (21.5)	81 (6.4)	981 (77.4)	239 (18.9)	47 (3.7)	778 (59.9)	400 (30.8)	121 (9.3)	2672 (69.7)	911 (23.8)	249 (6.5)
Washing hands after going out	1163 (91.9)	84 (6.6)	19 (1.5)	1106 (87.3)	128 (10.1)	33 (2.6)	1110 (85.5)	167 (12.9)	22 (1.7)	3379 (88.2)	379 (9.9)	74 (1.9)
Washing hands before eating	1228 (97.0)	33 (2.6)	5 (0.4)	1173 (92.6)	71 (5.6)	23 (1.8)	1227 (94.5)	56 (4.3)	16 (1.2)	3628 (94.7)	160 (4.2)	44 (1.1)
Washing hands after toileting	1217 (96.1)	37 (2.9)	12 (0.9)	1141 (90.1)	95 (7.5)	31 (2.4)	1214 (93.5)	64 (4.9)	21 (1.6)	3572 (93.2)	196 (5.1)	64 (1.7)
Washing hands after working	1207 (95.3)	50 (3.9)	9 (0.7)	1151 (90.8)	100 (7.9)	16 (1.3)	1214 (93.5)	69 (5.3)	16 (1.2)	3572 (93.2)	219 (5.7)	41 (1.1)
Washing hands with running water	1027 (81.1)	115 (9.1)	124 (9.8)	1160 (91.6)	60 (4.7)	47 (3.7)	1163 (89.5)	75 (5.8)	61 (4.7)	3350 (87.4)	250 (6.5)	232 (6.1)
Washing hands at least 20S	673 (53.2)	382 (30.2)	211 (5.5)	596 (15.6)	393 (10.3)	278 (7.3)	713 (18.6)	394 (10.3)	192 (5.0)	1982 (51.7)	1169 (30.5)	681 (17.8)

**Table 6:** Logistic regression analysis for influencing factors of handwashing with soap (n = 3832).

Variables	Total n (%)	Washing Hands with Soap n (%)	Univariate		Multivariate		
			OR (95% CI)	*p*-Value	*β*	OR (95% CI)	*p*-Value
**Gender**							
Male	1898 (49.5)	1301 (68.5)	0.895 (0.780–1.027)	0.114			
Female	1934 (50.5)	1371 (70.9)	1				
**Age (years)**							
15–34	689 (18.0)	501 (72.7)	1.508 (1.233–1.845)	<0.001	0.220	1.246 (0.969–1.603)	0.087
35–59	1812 (47.3)	1321 (72.9)	1.522 (1.307–1.773)	<0.001	0.342	1.408 (1.186–1.672)	<0.001
≥60	1331 (34.7)	850 (63.9)	1			1	
**Education level**							
Illiterate or have little literacy	1019 (26.6)	636 (62.4)	0.500 (0.397–0.630)	<0.001	–0.462	0.630 (0.478–0.831)	0.001
Primary school	890 (23.2)	608 (68.3)	0.650 (0.512–0.825)	<0.001	–0.319	0.727 (0.553–0.955)	0.022
Middle school	1340 (35)	980 (73.1)	0.820 (0.654–1.030)	0.088	–0.132	0.877 (0.683–1.125)	0.300
High school and above	583 (15.2)	448 (76.8)	1			1	
**Marital status**							
Unmarried	203 (5.3)	147 (72.4)	1.610 (1.098–2.362)	0.015	–0.145	0.865 (0.538–1.391)	0.550
Married	3316 (86.5)	2331 (70.2)	1.452 (1.142–1.845)	0.002	0.112	1.118 (0.864–1.447)	0.397
Divorced/widowed	313 (8.2)	194 (61.9)	1			1	
**Occupation**							
Farmer (worker)	2163 (56.4)	1482 (68.5)	1.023 (0.867–1.206)	0.788	0.052	1.054 (0.870–1.277)	0.593
Employees of governmental departments and retirees	193 (5.0)	151 (78.2)	1.690 (1.169–2.443)	0.005	0.335	1.398 (0.939–2.083)	0.099
Business and service workers	466 (12.2)	344 (73.8)	1.325 (1.034–1.699)	0.026	0.183	1.201 (0.915–1.577)	0.186
Students	78 (2.0)	61 (78.2)	1.687 (0.968–2.938)	0.065	0.389	1.475 (0.741–2.935)	0.268
Unemployed	932 (24.3)	634 (68.0)	1			1	
**Province**							
Shandong	1266 (33.0)	913 (72.1)	1.732 (1.468–2.044)	<0.001	0.587	1.799 (1.506–2.150)	<0.001
Shanxi	1267 (33.1)	981 (77.4)	2.297 (1.934–2.729)	<0.001	0.846	2.331 (1.946–2.790)	<0.001
Yunnan	1299 (33.9)	778 (59.8)	1			1	

**Table 7:** Logistic regression analysis for influencing factors of proper handwashing (n = 3832).

Variables	Total n (%)	Proper Hand Washing n (%)	Univariate		Multivariate		
			OR (95% CI)	*p*-Value	*β*	OR (95% CI)	*p*-Value
**Gender**							
Male	1898 (49.5)	699 (36.8)	0.904 (0.794–1.031)	0.132			
Female	1934 (50.5)	758 (39.2)	1				
**Age (years)**							
15–34	689 (18.0)	309 (44.8)	1.913 (1.581–2.315)	<0.001	0.250	1.284 (1.018–1.619)	0.035
35–59	1812 (47.3)	751 (41.5)	1.665 (1.433–1.935)	<0.001	0.304	1.356 (1.150–1.599)	<0.001
≥60	1331 (34.7)	397 (29.8)	1			1	
**Education level**							
Illiterate or have little literacy	1019 (26.6)	290 (28.5)	0.405 (0.327–0.500)	<0.001	−0.590	0.554 (0.431–0.712)	<0.001
Primary school	890 (23.2)	300 (33.7)	0.517 (0.418–0.641)	<0.001	−0.455	0.635 (0.498–0.809)	<0.001
Middle school	1340 (35)	578 (43.1)	0.772 (0.635–0.938)	0.009	−0.148	0.863 (0.696–1.070)	0.179
High school and above	583 (15.2)	289 (49.6)	1			1	
**Marital status**							
Unmarried	203 (5.3)	88 (43.4)	2.053 (1.414–2.980)	<0.001	−0.132	0.876 (0.549–1.399)	0.580
Married	3316 (86.5)	1284 (38.7)	1.695 (1.309–2.195)	<0.001	0.192	1.212 (0.922–1.593)	0.169
Divorced/widowed	313 (8.2)	85 (27.2)	1			1	
**Occupation**							
Farmer (worker)	2163 (56.4)	793 (36.7)	1.307 (1.109–1.541)	0.001	0.219	1.245 (1.051–1.476)	0.011
Employees of governmental departments and retirees	193 (5.0)	108 (56.0)	2.164 (1.721–2.720)	<0.001	0.793	2.210 (1.578–3.095)	<0.001
Business and service workers	466 (12.2)	228 (48.9)	2.870 (2.091–3.938)	<0.001	0.542	1.719 (1.355–2.181)	<0.001
Students	78 (2.0)	42 (53.9)	2.635 (1.653–4.201)	<0.001	0.860	2.362 (1.317–4.239)	0.004
Unemployed	932 (24.3)	286 (30.7)	1			1	
**Province**							
Shandong	1266 (33.0)	498 (39.3)	1.043 (0.890–1.222)	0.604			
Shanxi	1267 (33.1)	461 (36.4)	0.920 (0.784–1.080)	0.307			
Yunnan	1299 (33.9)	498 (38.3)	1				

## Data Availability

The datasets used and/or analyzed during the current study is available from the corresponding author on reasonable request.
